# Agri-Food By-Products as Ingredients: Exploring Purchase Intentions Among a Sample of Italian Consumers

**DOI:** 10.3390/foods14152664

**Published:** 2025-07-29

**Authors:** Maria Di Cairano, Nicola Condelli, Angela Lomonaco, Fernanda Galgano

**Affiliations:** Department of Agricultural, Forestry, Food and Environmental Science, University of Basilicata, Via dell’Ateneo Lucano 10, 85100 Potenza, Italy; nicola.condelli@unibas.it (N.C.); angela.lomonaco003@unibas.it (A.L.); fernanda.galgano@unibas.it (F.G.)

**Keywords:** by-products, consumer attitude, food neophobia, PLS-SEM, purchase intention, upcycled foods

## Abstract

Consumer perceptions of upcycled foods, as well as the determinants of food choices, are still not well understood. The aim of this study was to evaluate the impact of psychological and personal traits on purchase intention (PI) towards upcycled foods of Italian consumers. Additionally, willingness to try (WTT), willingness to buy (WTB), and willingness to pay (WTP) for yogurt, bread, and biscuits made with by-products, namely, hazelnut skin and grape pomace powder, were collected. A web-based survey involving 505 consumers was conducted, and partial least squares structural equation modeling (PLS-SEM) was used to evaluate the model. It resulted that food neophobia and attitude towards upcycled foods had a significant impact on PI. In turn, attitude was affected by food neophobia as well as by objective knowledge about food by-products. Unexpectedly, frugality and environmental concern did not affect PI. WTP was product-specific; when WTP was compared to a reference price, it was higher for the yogurt prototype, followed by the bread and biscuits prototypes. Food neophobia affected WTT, WTB, and WTP. This study contributes to providing new insights into the determinants of consumers’ purchase intention for upcycled foods, which are an emerging category of products.

## 1. Introduction

In the face of growing concerns about food waste and sustainability, upcycling agri-food by-products into ingredients has emerged as a promising approach to reduce food losses and promote a more sustainable food system [1,2]. Upcycling involves transforming ingredients that otherwise would not have gone to human consumption into new and valuable products, extending the life cycle of food resources, and minimizing their environmental impact.

To date, a universal definition of upcycling still does not exist. A first attempt to provide a definition of upcycled food products was made in 2020 by a team of American researchers [3] interviewing food manufacturers affiliated with Upcycled Food Association. The study led to a definition comprising the aspect relating to the elevation of food that would be wasted, as well as the benefits for the environment and society. More recently, the concept of upcycling has been further elucidated. Indeed, Aschemann-Witzel et al. [4] provided a comprehensive definition of upcycled foods, distinguishing between “alternative use sense” and “novel use sense”: “The first is upcycling in an ‘alternative use sense,’ because it is about food or ingredients that could as well be eaten and are rescued from the threat of disposal, upcycling it in one way or other into alternative foods, and contributing value to society through avoidance of food being wasted. The second is upcycling in a ‘novel use sense.’ The difference to the first is that it starts with ingredients that are not regarded or commonly seen as edible, but by upcycling these in one way or other, results in new foods” [4]. Upcycling can involve food by-products; by repurposing these by-products into ingredients, manufacturers can create value from what was previously considered waste.

Upcycling can lead to three advantages, akin to the three pillars of sustainability [2]: economical, by giving new value to recovered raw materials cheaper than the original; environmental, by reducing the pressure on the ecosystem; and on a social level, by reducing food waste. In addition, food by-products, when used as food ingredients, may act as functional ingredients [5,6]. Indeed, they could represent a source of fiber, protein, and many other bioactive compounds.

The market of upcycled food is young, and foods obtained from upcycled raw materials are not widespread. Anyway, a growing number of research studies highlight the importance of the topic; additionally, market agencies foresee a robust growth in the next 10 years for the upcycled food product market [7,8]. Different companies, both new and existing ones, are focusing their efforts on the production of upcycled foods or ingredients, emphasizing in their campaigns the importance of their approach to the environment.

The acceptance of these products is intended as the intention to purchase them, and the liking of the product itself should not be taken for granted. Indeed, different dimensions are involved in the acceptance of this new category of foods, such as consumers’ characteristics, marketing strategies, political measures, as well as the product itself.

The topic is complex, and consumers’ preferences and behaviors towards upcycled foods are still unclear. To date, different studies have tried to explore factors affecting them. An aspect not to be neglected is that when products are made from recycled materials, consumers may perceive them as of lower value [9]. Moreover, the acceptance of the use of by-products in food production is complex due to consumers’ traits such as food neophobia and technophobia [10]. Aschemann-Witzel and Stangherlin [9] collected, from published papers (2010–2020), the factors that influence consumers’ acceptance of waste-to-value products, classifying them as “individual factors”, “context factors”, and “product-related factors”. These factors and their interaction can shape consumer acceptance of waste-to-value food products. For example, Grasso et al. [11] compared consumers’ attitudes of China and the US towards upcycled foods, highlighting differences in the attitudes and preferences among the two groups; Stelick et al. [12] reported that providing information on sustainability affected the PI of bars with brewers’ spent grains more than nutritional information, whereas Zhang et al. [13] reported that upcycled food quality is perceived differently by different consumer generations. Other researchers from Italy [14] highlighted that sense of retribution, general health interest, and moral domains could be linked to PI of upcycled foods. These are just some examples underlining the complexity of the topic. Therefore, it is crucial to understand the determinants of consumer PIs and attitudes towards upcycled foods. This new category of products and all the related topics represent something that producers, consumers, and governments began to address in the last few years and will increasingly confront in the near future. Therefore, this study aims to explore the relationships between several psychological traits and the purchase intention for foods containing agri-food by-products. To this end, a theoretical model was tested on a sample of Italian consumers to elucidate the key determinants of intention and willingness to pay for such products.

To ensure clarity, the questionnaire specifically addressed the use of by-products as ingredients rather than adopting the term “upcycled foods”. In fact, upcycled food does not have a direct Italian translation; the closest equivalent is “cibi riciclati” (recycled foods), which may evoke negative or misleading connotations. This issue is not unique to Italian. For instance, in the Norwegian context, the absence of a direct and widely accepted translation for upcycled food has been noted [15]. Similarly, in Italian, the terminology is still evolving and lacks a universally adopted equivalent. This work contributes to broadening the information on the consumers’ attitudes towards upcycled foods.

## 2. Materials and Methods

### 2.1. Sample, Data Collection, and Measurement

The questionnaire was addressed to the Italian adult population, and participants were recruited by snowball sampling, a non-probability sampling technique. It was spread by means of mailing lists, social networks, instant messaging apps, and word of mouth. The questionnaire was built and distributed by means of Microsoft Forms (Microsoft 365, Microsoft, Redmond, WA, USA). Data were collected between March and July 2023.

The questionnaire measured the impact of attitude, food neophobia, environmental concern, green practices, frugality, and previous knowledge on the PI of foods obtained from by-products of the agri-food industry. Additionally, three examples of upcycled food products were proposed to evaluate consumers’ willingness to try, to buy, and to pay (WTT, WTB, and WTP). All measurements were made on a 7-point Likert scale (1 = strongly disagree, 7 = strongly agree), except for attitude (7-point bipolar scale), green practices (5-point Likert frequency scale), and objective knowledge (true/false questionnaire).

The various constructs were drawn, or adapted, from previous works in the literature. Table 1 illustrates the structure of the questionnaire, the scale employed for each construct, and the references.

The first part of the questionnaire included an introduction to the survey and informed consent. Participants were informed about the estimated completion time, the aim of the study, risks, and benefits, and that all data would be de-identified and only reported in aggregated form. Participants who did not give their consent were not able to proceed with completing the questionnaire.

Then, questions to measure 7 psychometric characteristics (food neophobia, environmental concern, green practices, frugality, subjective and objective knowledge, and attitude towards the use of food by-products as ingredients) were submitted to consumers. A short text including a description of by-products (Supplementary Material File S1) was provided before attitude constructs. This was deemed necessary to ensure a baseline understanding of the topic, given the expected low consumer familiarity, and to avoid responses based on misunderstanding. Subsequently, a question oriented to collect the PI of foods containing by-products as ingredients was submitted to consumers. The question was not on a specific food product but on the broader category of “foods containing by-products as ingredients”. Then, the last part of the questionnaire was used for collecting willingness to try, to buy, and to pay for three examples of foods containing by-products. Food product examples were taken from scientific literature, among the categories of products that are generally consumed by the Italian population. A dairy product and two baked goods were considered; namely, yogurt with hazelnut skin [23], bread with grape pomace powder [24], and biscuits with brewers’ spent grains [25] were selected. Concerning WTP, consumers were asked to indicate the price they would be willing to pay for the products while provided with an external reference price that was the medium selling price for the “conventional” version of each product. The medium price provided for each product was calculated as the mean of prices in all Italian provinces, reported by the Ministry of Enterprises and Made in Italy on its price observatory for consumer goods, as of November 2022. At the very end of the questionnaire, demographic information was collected.

#### Subjects

The questionnaire was checked and revised by the Transparency Office and Data Protection Officer of the University of Basilicata to verify compliance with European data protection legislation (Reg EU 2016/679). Due to the negligible risk posed to participants, no formal ethical approval was required. Participants gave their consent to participate prior to completing the questionnaire. The study was conducted in accordance with the ethical principles outlined in the Declaration of Helsinki and the European Code of Conduct for Research Integrity.

### 2.2. Theoretical Framework and Hypothesis Development

**Figure 1:**
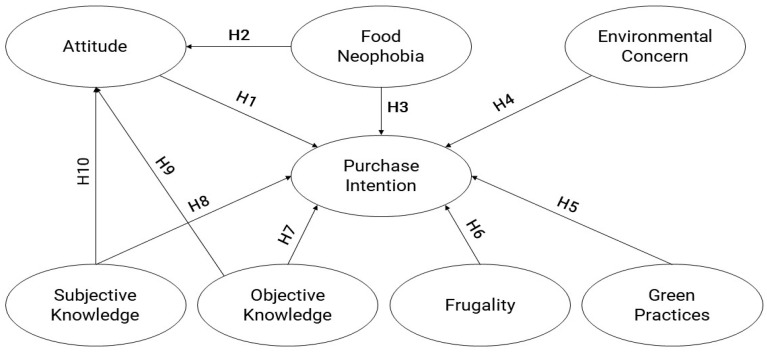
Research framework.

The following paragraphs briefly illustrate the hypotheses and their background, whereas Figure 1 summarizes the conceptual model of the research.

#### 2.2.1. Attitude

Attitude refers to the degree to which a person has a favorable or unfavorable evaluation of a specific behavior. Previous research has shown that a positive attitude towards specific products, such as organic milk [26], organic meat [27], plant-based alternatives to yogurt [28], insects [29], and expired products that are still edible [30], positively influences consumers’ PI towards them. Similarly, a positive attitude towards functional foods was found to be a key driver of the consumption of foods containing grape pomace powder [31]. Based on the above, it was hypothesized that:

**H1.**  *A positive attitude towards the use of food by-products as food ingredients has a positive impact on consumers’ PI towards them.*

#### 2.2.2. Food Neophobia

Food neophobia is the reluctance to eat new foods or their avoidance [17]. Together with disgust, it may hinder the acceptability of new food products due to aversion to sensory characteristics, danger, fear of the consequences of eating unfamiliar foods, or disgust arising from ideas about the nature or origin of the food [31]. Consumers may reject or be skeptical of “recycled” foods because of their fear of new or unfamiliar [32,33,34]. Therefore, food neophobia is expected to be a barrier to the purchase of food products containing by-products of the agri-food industry, as well as the attitude towards them. Hence, it was hypothesized that:

**H2.**  *Food neophobia has a negative impact on consumers’ attitudes towards the use of by-products in food formulations.*

**H3.**  *Food neophobia negatively influences consumers’ PI for food products containing by-products of the agri-food industry.*

#### 2.2.3. Environmental Concern

Environmental concern refers to the degree to which consumers are aware of and concerned about environmental problems, and their willingness to take action to address them [35]. Previous research has shown that environmental concern has a significant positive impact on PIs for veggie burgers [36] and eco-friendly products [37]. This is likely because consumers who are more concerned about the environment are more prone to purchase foods that are produced in a sustainable way. Indeed, it was previously reported that a higher PI of functional foods with pomace powder in consumers with a higher degree of concern for the environment [31]. Based on this evidence, it was hypothesized that:

**H4.**  *Environmental concern positively influences consumers’ PI for food products containing by-products of the agri-food industry.*

#### 2.2.4. Green Practices

Green practices are everyday actions that are taken to protect the environment, such as energy saving, recycling, waste reduction, and sustainable transportation [38]. Research has shown that consumers who engage in more green practices have greater satisfaction with and more frequent purchases of green products [38]. White, Habib, and Hardisty [39] suggested a positive spillover effect in green consumer behavior, where adopting one environmentally friendly practice increases the likelihood of adopting others. Hence, it could be expected that consumers who engage in more daily actions to protect the environment will also be more likely to purchase food products containing by-products of the food industry. This is because upcycling by-products into food ingredients is a way to reduce waste and promote environmental sustainability. Thus, it was hypnotized that:

**H5.**  *Green practices positively influence consumers’ PI for food products containing by-products of the agri-food industry.*

#### 2.2.5. Frugality

Frugality is the behavior of reducing the waste of resources, including money, and making use of what one has. It can lead to reduced consumption and the implementation of reuse, repair, and recycling behaviors [40]. Several authors have pointed out that frugality should not be confused with avarice [41,42]. Instead, it is a voluntary restriction on consumption for the efficient use of resources and the avoidance of waste [20], which can drive more sustainable consumption choices.

Upcycled foods may fall within the concept of frugality. Aschemann-Witzel et al. [20] reported that people with a more frugal orientation have a more positive attitude towards this type of food. It is therefore likely that consumers with frugal traits are more prone to purchase foods that avoid the waste of resources. Based on the above, the following hypothesis was proposed:

**H6.**  *Frugality has a positive impact on consumers’ PI for foods containing by-products of the agri-food industry.*

#### 2.2.6. Product Knowledge—Objective and Subjective Knowledge

Subjective knowledge is what an individual believes he knows about a topic or product, while objective knowledge is based on the accurate understanding of the information stored in memory. Product knowledge, even in the absence of direct experience, can lead to the formation of a specific attitude based on the information and knowledge that the consumer has. Product knowledge influences consumer attitudes because it allows them to better understand the products and think rationally about them. For example, consumers who are well informed about renewable products are better able to understand that these can save resources and energy than those who are not [43].

Regarding food products, both objective and subjective knowledge have been found to influence the intention to purchase insect-based products [29]. Baldissera et al. [31] also reported that greater prior knowledge of functional foods and whole grain products translates into a higher intention to purchase products obtained with grape pomace powder. Diverging information has been reported on the role of previous knowledge on the PI of and attitude towards organic food products. Nautiyal and Lal [21] found that objective knowledge has a crucial role in shaping Indian consumers’ purchasing intentions for organic products, unlike subjective knowledge, which has no significant effect. Similarly, Bhutto et al. [27] reported that knowledge of organic production practices did not have a significant effect on the development of intention to purchase organic meat. Based on the above considerations, it was hypothesized that:

**H7.**  *Objective knowledge of food industry by-products positively influences consumers’ PI for foods containing agri-food by-products as ingredients.*

**H8.**  *Objective knowledge of food industry by-products positively influences consumer attitude towards the use of by-products in food formulations.*

**H9.**  *Subjective knowledge of topics related to food industry processing by-products has a positive impact on consumers’ PI for foods with agri-food by-products as ingredients.*

**H10.**  *Subjective knowledge of topics related to food industry processing by-products has a positive impact on the PI of foods with agri-food by-products as ingredients.*

### 2.3. Data Analysis

Structural Equation Modeling (SEM) technique was used to test the theoretical model with the software Smart PLS (v. 4.0.9.6, SmartPLS GmBH, Bönningstedt, Germany) [44]. Measurement model assessment included the evaluation of construct reliability and validity. Construct reliability was established through Cronbach’s Alpha and Composite Reliability. Convergent validity was assessed through average variance extracted (AVE), whereas discriminant validity was assessed through Fornell–Larcker Criterion and heterotrait–monotrait (HTMT) ratio of the correlations. The causal relationship presumed was tested through analysis of the structural model. Subsequently, bootstrapping with 5000 subsamples was used during hypothesis testing [45].

To test for the potential moderating effects of gender and age, a multi-group analysis (MGA) was performed. Path coefficients were estimated separately for each subgroup via bootstrapping (5000 resamples), and the differences between the coefficients were assessed for statistical significance using a *t*-test.

To further investigate the hierarchical relationship among behavioral intentions, a Path Analysis was implemented within the PLS-SEM framework. Three separate models, one for each product example, were estimated to test the sequential causal chain PI→WTT→WTB→WTP. The statistical significance of the path coefficients was determined via bootstrapping (5000 resamples).

One-Way ANOVA (Analysis of Variance), followed by Tukey HSD test (*p* < 0.05), was used to compare both WTT and WTB mean values of yogurt, bread, and biscuits with by-products. Student-*t* test (*p* < 0.05) was conducted to evaluate differences between WTT and WTB for each sample. Moreover, respondents were clustered based on their food neophobia (neophilic, neutral, and neophobic); a non-parametric Kruskal–Wallis test, followed by Dunn test (*p* < 0.05), was used to compare WTT, WTB, and WTP mean values expressed by each cluster. These tests were carried out with XLSTAT Premium Version (2019.4.2, Addinsoft, Paris, France) in Excel 365 (Microsoft 365; Microsoft, Redmond, WA, USA).

## 3. Results and Discussion

### 3.1. Characteristics of the Sample

The survey was submitted to the Italian adult population by means of an online questionnaire: 534 people answered the questionnaire, 13 did not give consent, whereas 16 answers were removed due to the very short time (<6 min) employed to answer the questionnaire; 505 answers were considered for data analysis. Sociodemographic information of the sample is reported in Table 2. The utilized sampling technique led to a slight overrepresentation of females (62.57%) and younger age groups (approximately 54% of respondents were under 34 years old, while about 18% were over 55 years old).

It should be noted that this is an explorative study and does not claim to be generalizable to the entire Italian population. Younger respondents (18–34) are strongly overrepresented, while individuals over 65 years of age, who account for approximately 24% of the Italian population [46], make up only 4.4% of the sample. Similarly, students and student–workers account for nearly 40% of the sample, which is not reflective of national employment statistics. The study employed a non-probability sampling technique, and data were collected exclusively through online channels. This approach likely favored the participation of younger and more digitally literate individuals, leading to a lower response rate among older consumers. While we recognize that other methods might have yielded a more representative sample, resource constraints prevented the implementation of such an approach.

### 3.2. Descriptive Statistics, Reliability, and Validity Tests

An analysis of the descriptive statistics (Supplementary Material File S2) was conducted to assess the variability in responses. Responses had sufficient variability to discriminate among consumers. Nevertheless, the results show two patterns: On the one hand, constructs, such as Environmental Concern (EC) and Frugality (FRU), exhibited low variability, indicating a stronger consensus compared to other items. This could be potentially influenced by social desirability. On the other hand, constructs representing individual traits, such as Food Neophobia (NP) and Knowledge (SK, OK), showed high variability. This heterogeneity confirms that these variables effectively discriminate between respondents. Also, the crucial constructs of Attitude and Purchase Intention displayed sufficient variability, justifying the use of PLS-SEM to model their relationships.

The measurement model was evaluated to ensure the reliability and validity of the constructs. Hair et al. [47] suggested desirable factor loadings of over 0.708, with a minimum acceptable value of 0.50. Anyway, it is recommended to check the impact of removing items with loadings below 0.7 on composite reliability and Average Variance Extracted (AVE) before automatic removal [41,42]. Acceptable AVE is at least 0.500, indicating that the construct explains at least 50% of its items’ variance [48].

All factor loadings in the current study (Supplementary Material File S2) were above 0.5. However, items GP1 (0.540) and GP2 (0.631) were removed because the initial AVE value was below 0.5. Their removal increased AVE to 0.539. Specifically, the items removed from the Green Practices construct were “I buy products with recyclable packaging” (GP1) and “I recycle plastic, paper, and glass” (GP2). Although related to environmentalism, these two items describe habitual, low-effort behaviors that are often dictated by local regulations. Similarly, item NP2 with a slightly lower loading (0.572) was removed since its removal had a positive impact on AVE (0.5 vs. 0.486). While fully acknowledging the validated and international status of the Food Neophobia Scale, the item NP2, “In the choice of food I do not trust novelty”, showed weaker statistical performance. This may depend on a linguistic difference: The concept of not trusting can capture a broader dimension of skepticism, which is distinct from the fear or avoidance that is core to neophobia. Other items with outer loadings slightly lower than 0.7 were retained because overall construct reliability and validity were higher than the recommended threshold values. Table 3 summarizes reliability and validity values after the removal of the above-mentioned items.

Reliability was assessed using Cronbach’s alpha, rho_a, and rho_c. The Cronbach’s alpha of each construct, except for GP, was greater than 0.708, suggesting the reliability of the scales. Even though GP’s Cronbach’s alpha was slightly lower than the recommended threshold, its composite reliability exceeded HTMT and the Fornell–Larcker criterion [44]. Discriminant validity was established through cross-loadings analysis (Supplementary Material File S2).

### 3.3. Structural Model Analysis

Path analysis was conducted to test the hypothesis; the bootstrap resampling method with 5000 resamples was employed to assess the path significance levels.

Overall, the model was able to explain a substantial portion of the variance in purchase intention (R^2^ = 0.555). The summary of hypothesis testing results is reported in Table 4.

A preliminary overview of the path coefficients indicates that the primary drivers in the model are psychological and knowledge-based. Attitude emerged as the most powerful predictor of PI and was itself strongly influenced by Objective Knowledge and Food Neophobia. Conversely, paths originating from Environmental Concern and Frugality, as well as Subjective Knowledge, are weak and not statistically significant. This finding offers a strategic insight for managers and policy makers: Communication efforts based on these general values are unlikely to be effective levers for driving purchase intention.

Attitude had a significant positive impact on the intention to purchase foods containing by-products as ingredients (H1). This aligns with previous research highlighting the significant role of consumer attitudes in influencing purchase decisions [45], and it underscores the importance of consumer attitudes in shaping their purchasing decisions. Though showing a good variability, the mean scores for attitude in our sample were noticeably positive. This generally favorable predisposition is consistent with aspects of Italian culture, where the no-waste principle is a deep-rooted social norm inherited from past generations.

Food neophobia, on the other hand, had a significant negative impact on attitude and PI (H2, H3). The result is consistent with previous research [46,47]; food neophobia is a barrier to the acceptance of new foods, including foods containing upcycled ingredients. In a recent scoping review [46], the factors affecting consumers’ acceptance of upcycled food products were investigated; the authors highlighted a strong relationship between neophobic tendencies and acceptance of upcycled food products. The individual trait of food neophobia is particularly interesting when interpreted in the context of Italian culinary norms. Italy is characterized by a strong culinary tradition and a deep attachment to specific recipes and ingredients. This polarization makes neophobia a crucial psychological trait to consider when introducing food innovations into a market with such a deep-rooted gastronomic identity. Contrary to the tested hypothesis (H4), environmental concern was not found to have a significant impact on PI. A possible explanation is that participants may not yet recognize a direct link between purchasing foods containing upcycled ingredients and reducing environmental impact. Furthermore, this mismatch between the concern and the behavior (in this case, the statement of purchase intention) may reflect the “attitude–behavior gap” [49], with consumers declaring to be concerned about the environment but struggling to translate this concern into concrete action [50].

Green practices, which somehow can be seen as a counterpart of environmental concern, showed a significant effect on PI (H5). The more consumers engage in practices aimed at protecting the environment, the higher is PI for upcycled foods. Although the path coefficient is modest (0.086), it does not indicate a lower importance of the construct; it suggests that existing green behaviors, while not the primary driver, can act as an enabling factor for this type of purchase, mediated or moderated by other factors. As a practical implication, this suggests that consumers who actively engage in sustainable practices could represent a key initial and receptive target segment for early marketing and communication efforts.

The concept of frugality in the context of upcycled food production is quite new [20]. The authors found that when frugality framing was used in the experiment, the attitude towards upcycled foods was higher. In this work, frugality did not have a significant impact on PI (H6). As per the EC construct, this seems an apparent contradiction, especially for a sustainable product, but it could be explained by the value–action gap [51]. Consumer behavior literature suggests that general and abstract “values” often fail to translate into specific behaviors. Other factors, such as the specific attitude towards the product or food neophobia, among others, carry greater weight, leaving background values behind when it comes to making a decision on the product [49].

Objective knowledge had a significant effect on attitude towards the use of by-products as food ingredients (H7). Indeed, it has been previously seen that a previous knowledge of a topic contributes to shaping consumers’ attitudes [21,23,38]. Nevertheless, the direct path from objective knowledge to PI (H8) was not significant. This suggests a mediation effect of attitude between OK and PI. Hence, a new hypothesis was added to the original framework (H11) about the mediating role of attitude in the relationship between objective knowledge and purchase intention. The results supported full mediation. Subjective knowledge (H9), which is what one thinks to know about a specific topic, did not have a significant effect on attitude and PI (H10). In this study, what consumers think they know about the topic seems not to be relevant to their decision-making process.

To deepen the understanding of the model, a Multi-Group Analysis (MGA) was conducted to examine whether the hypothesized causal relationships differed significantly across key demographic characteristics, namely, gender and age MGA revealed a moderating effect of gender on several key paths of the model: Specifically, the impact of food neophobia on attitude (NP→ATT, difference F-M: −0.144, *p* < 0.05), that of subjective knowledge on attitude (SK→ATT, difference F-M: −0.280, *p* < 0.01), and the link between attitude and purchase intention (ATT→PI, difference F-M: −0.136, *p* < 0.05) were found to be statistically different between men and women. Age, conversely, showed a moderating role in the relationship between attitude and purchase intention (ATT→PI, difference “18–44”–“45+”: −0.138, *p* < 0.05) only for the older consumer segment.

### 3.4. Willingness to Try, Buy, and Pay

To investigate consumer attitudes towards specific food products containing by-products, three products were presented to consumers, and their WTT, WTB, and WTP were collected. Namely, a short description of a yogurt with hazelnut skin, a bread containing grape pomace powder, and biscuits with brewers’ spent grains was presented to consumers.

Significant differences were found between mean WTT and WTB expressed for each product (Table 5), indicating that consumers are more prone to express their intention to try the new products but not to buy them. Indeed, consumers may be more willing to try new products without committing to a purchase, to avoid buying something that could disappoint them. However, no significant differences (*p* > 0.05) were observed in WTB or WTT among the three products (Table 5).

Over 65% of consumers expressed a positive WTB and WTT for the proposed products, scoring above 4 on a 7-point Likert scale. However, WTB scores were slightly lower, with scores above 4 ranging from 53.66% to 59.01%. While positive responses dominated, a portion of consumers expressed the lowest possible score (1 = not at all) on the WTT scale for each product: 7.92% for yogurt, 12.08% for bread, and 9.90% for biscuits.

While most consumers appear receptive to trying food products with by-products (over 65% scored positively in our study), a portion did not want to try at all (between 7.92% and 12.08%), likely due to unfamiliarity or specific personal traits. Actions aimed at educating consumers about the benefits of these products, such as sustainability and potential health benefits, could be crucial for a wider acceptance [46]. While WTT and WTB values were quite encouraging, WTP values showed a slightly different trend. In this study, WTP for yogurt, bread, and biscuits made with by-products was collected via an open-ended question. Consumers were asked how much they would be willing to pay for each product presented while provided with the average market price of a similar conventional product as a reference point. Consumers expressed a different WTP based on the product presented; Table 6 reports the mean WTP values expressed by consumers and the difference with the reference price for the conventional products.

On average, consumers are willing to pay more for the yogurt with hazelnut skin compared to the reference price provided, but less for bread with grape pomace powder and biscuits with brewers’ spent grains. It is important to note that each example product combines a unique food matrix with a specific by-product. As such, it is not possible to determine whether consumers’ willingness to pay was influenced more by the nature of the food product (e.g., yogurt vs. bread) or by the type of by-product (e.g., hazelnut skin vs. grape pomace). Nonetheless, some methodological precautions were taken to reduce potential bias. First, all the selected food matrices—yogurt, bread, and biscuits—are widely recognized and regularly consumed in Italy, which reduces the influence of unfamiliarity on participants’ responses. Second, a reference price was provided for each product. This allowed respondents to evaluate the price for products containing by-products against a realistic baseline. As reported above, the collected data do not provide any information on the drivers of WTP, which could depend on both the product category and the type of by-product added. It could be supposed that consumers may be willing to pay more for a product with a by-product/ingredient they are already familiar with or have eaten at home (e.g., hazelnuts with their skin on). On the other hand, consumers may be unwilling to pay more for staple products such as bread and biscuits, or for products containing something that is usually wasted, or they are not familiar with, such as grape pomace powder and brewers’ spent grains. In a recent study, Grasso et al. [11] reported that some food categories are preferred by consumers as food, where by-products can be added. For example, breakfast, snack, pasta, and soup were the top choices in the United States, whereas breakfast, snack, dairy, and baked goods were the top choices in China. Additionally, the same study investigated the preferred combinations of by-products and food categories, showing that consumers can have different preferences, and some combinations of food category/type of by-product are preferred compared to others. Generally, WTP for upcycled foods is lower than WTP for conventional foods, but product-specific differences could exist [49,50]. Hence, differences in WTP could be due to consumers who are more likely to accept upcycled alternatives for some food categories than others, as previously reported by Bhatt et al. [52]. In this regard, Ghazanfar et al. [53] reported that consumers’ WTP was significantly less for upcycled granola bars, chicken nuggets, and ice cream but not for muffins and pasta sauce. Additionally, there is also a deep-rooted idea that products containing by-products should cost less compared to the conventional ones. In fact, by-products are often considered to be waste materials, and consumers may believe that these products should be priced lower as a result. Nonetheless, it is not known that, from an economic perspective, in many cases, wasting by-products is economically more advantageous than saving them [26]. As expected, when clustering consumers based on their food neophobia, consumers with a higher level of food neophobia scored lower (*p* < 0.05) for WTT and WTB for all three products considered. Additionally, food neophobia level affected WTP of bread and biscuits, with neophobic consumers scoring low for WTP of bread and biscuits (*p* < 0.05). Nevertheless, no significant differences were observed among the three groups for yogurt; this result could depend on the “low” external reference price provided. When analyzing WTT and WTB data for each product and within each food neophobia level, this result was not confirmed. Indeed, neophobic consumers scored lower for WTT and WTB for yogurt as well as for bread and biscuits. This indicates that food neophobia level affected these parameters regardless of the product considered.

#### Path Analysis of Behavioral Intentions

The Path Analysis, conducted to explore the sequential chain of behavioral intentions, showed consistent results across all three product prototypes. As reported in Table 7, all causal paths in the PI→WTT→WTB→WTP model were found to be positive and statistically significant (*p* < 0.001) in all three cases. Results showed that a general intention to purchase upcycled products (PI) effectively translates into a willingness to try (WTT), and that, in our hypothetical scenario—without the real tasting of the product—a strong WTB is generated. This latter link is particularly strong across all products.

## 4. Conclusions

This study contributes to consumer science by demonstrating that for novel food categories like upcycled products, psychological traits are more critical than general pro-environmental values. Specifically, it was found that food neophobia and consumer attitude are primary drivers of the purchase intention for foods containing agri-food by-products, whereas environmental concern did not show a statistically significant impact on PI. Furthermore, the perceived value of foods containing by-products as ingredients is not absolute but is product-dependent, with some prototypes able to command a price premium. These findings suggest that marketing strategies should focus on shaping favorable attitudes and be tailored to each product’s specific value proposition. However, it is important to note that these results reflect the responses of a minimally informed consumer group; we acknowledge that this single-group informed design limits the generalizability of the findings to an uninformed population.

## Figures and Tables

**Table 1:** Questionnaire structure, references, and scales used.

Sections	Items (*n*)	Scale	References
Introduction and informed consent			
Food neophobia (FN)	10	7-point Likert	[16,17]
Environmental concern (EC)	5	7-point Likert	[18]
Green practices (GPs)	5	5-point frequency	[19]
Frugality (FRU)	3	7-point Likert	[20]
Subjective knowledge (SK)	5	7-point Likert	Adapted from [21]
Objective knowledge (OK)	6	True or false|do not know	-
Information on by-products			
Attitude (ATT)	4	7-point bipolar adjectives	[22]
Purchase intention (PI)	1	7-point Likert	-
Willingness to try (WTT)		7-point Likert	-
Willingness to buy (WTB)		7-point Likert	-
Willingness to pay (WTP)		Open-ended question	-
Demographics		Multiple choice questions	

**Table 2:** Respondents’ profile.

Variable	Categories	Frequency	Frequency (%)
Gender	Male	189	37.43
	Female	316	62.57
Age	18–24	92	18.21
	25–34	182	36.04
	35–44	86	17.03
	45–54	53	10.46
	55–64	70	13.86
	>65	22	4.36
Employment	Student	140	27.72
	Student–worker	58	11.48
	Public employee	126	24.95
	Private employee	95	18.81
	Self-employed	45	8.91
	Retired	29	5.74
	Unemployed	12	2.38
Education	Middle school or lower	12	2.38
	High school	149	29.50
	Bachelor degree	116	22.97
	Master or equivalent	148	29.31
	PhD–other	80	15.84
Domicile city inhabitants	<5000	182	36.04
	5000–49,999	121	23.96
	>50,000	202	40.00

**Table 3:** Reliability and validity analysis.

	Cronbach’s Alpha	Composite Reliability (rho_a)	Composite Reliability (rho_c)	Average Variance Extracted (AVE)
ATT	0.907	0.909	0.935	0.782
EC	0.799	0.802	0.862	0.556
FRU	0.738	0.825	0.848	0.652
GP	0.607	0.639	0.787	0.539
FN	0.878	0.893	0.902	0.509
OK	0.849	0.860	0.887	0.568
SK	0.929	0.939	0.946	0.780

ATT, attitude; EC, environmental concern; FRU, frugality; GPs, green practices; FN, food neophobia; OK, objective knowledge; SK, subjective knowledge.

**Table 4:** Summary of hypothesis testing results.

	Path	Path Coefficient	Standard Deviation	T Statistics	*p* Values	Results
H1	ATT→PI	0.666	0.035	19.272	0.000	Supported
H2	NP→ATT	−0.234	0.037	6.134	0.000	Supported
H3	NP→PI	−0.117	0.034	3.424	0.001	Supported
H4	EC→PI	−0.005	0.032	0.284	0.776	Not supported
H5	GP→PI	0.086	0.035	2.365	0.018	Supported
H6	FRU→PI	−0.006	0.036	0.267	0.789	Not supported
H7	OK→PI	0.06	0.088	0.613	0.540	Not supported
H8	OK→ATT	0.69	0.111	6.156	0.000	Supported
H9	SK→PI	0.03	0.042	0.795	0.427	Not supported
H10	SK→ATT	0.073	0.055	1.33	0.183	Not supported
H11	OK→ATT→PI	0.204	0.038	5.47	0.001	Supported

PI, purchase intention; ATT, attitude; NP, food neophobia; EC, environmental concern; GPs, green practices; FRU, frugality; OK, objective knowledge; SK, subjective knowledge.

**Table 5:** Willingness to try (WTT) and to buy (WTB) for yogurt with hazelnut skin, bread with grape pomace powder, and biscuits with brewers’ spent grains.

	Willingness to Try	Willingness to Buy
Yogurt with hazelnut skin	5.25 ± 1.80 aA	4.81 ± 1.77 aB
Bread with grape pomace powder	5.12 ± 1.87 aA	4.70 ± 1.85 aB
Biscuits with brewers’ spent grains	5.24 ± 1.89 aA	4.86 ± 1.89 aB

Different lowercase letters along the column indicate differences among the samples; different capital letters along the row indicate differences in WTT and WTP for the same sample (*p* < 0.001).

**Table 6:** Willingness to pay for the proposed products in comparison to a reference price.

Product	Reference Price * (EUR)	Mean WTP (EUR)	Δ
Yogurt with hazelnut skin	0.95	1.04 ± 0.48	+9.5%
Bread with grape pomace powder	3.95	3.34 ± 1.22	−15.4%
Biscuits with brewers’ spent grains	4.16	3.66 ± 1.20	−12.0%

* Mean values of conventional product; data were collected from “Osservatorio prezzi al consumo” of the Ministry of Enterprises and Made in Italy (data November 2022).

**Table 7:** Path analysis of behavioral intentions.

Product	Path	Path Coefficient	Standard Deviation	T Statistics	*p* Values
Yogurt with hazelnut skin	PI→WTT	0.536	0.039	13.896	<0.001
	WTT→WTB	0.844	0.017	51.015	<0.001
	WTB→WTP	0.226	0.070	3.156	<0.001
Bread with grape pomace powder	PI→WTT	0.567	0.033	17.070	<0.001
	WTT→WTB	0.869	0.016	55.829	<0.001
	WTB→WTP	0.445	0.043	10.315	<0.001
Biscuits with brewers’ spent grains	PI→WTT	0.566	0.037	15.407	<0.001
	WTT→WTB	0.880	0.016	53.594	<0.001
	WTB→WTP	0.457	0.045	10.154	<0.001

## Data Availability

Raw data are not deposited in a public repository due to their potential use in subsequent publications and in adherence to the informed consent provided to participants.

## Supplementary Materials

The following supporting information can be downloaded at https://www.mdpi.com/article/10.3390/foods14152664/s1, File S1: Information on by-products; File S2: Table S1. Descriptive statistics of questionnaire items; Table S2. Results of measurement model evaluation—Reliability and validity analysis; Table S3. Cross loadings.
