# Supplementary material for: Agri-Food By-Products as Ingredients: Exploring Purchase Intentions Among a Sample of Italian Consumers
Source: Foods. 2025 Jul 29;14(15):2664. doi: 10.3390/foods14152664 (PMC12345695; doi:10.3390/foods14152664)
Supplement: Supplementary file 1 [file foods-14-02664-s001.zip › File S1.pdf]

Supplementary materials S1 - Information on by-products

Please read carefully the following:

By-products are substances generated from a production process whose primary purpose is not the production of such substance. These can be used during a production process by the same producer or by third parties. These substances must be able to be used directly without the application of treatments beyond normal industrial practice. Their reuse is legal if it does not have a negative impact on the environment or human health. The use of byproducts in Italy is regulated by Decree-Law 152/06, which defines a by-product based on the above conditions.

Byproducts of the food industry often become feed, fertilizers, or biofuels.

Examples of by-products include: corn gluten, vegetable oil extraction meals, beet pulp, corn germ, byproducts of the milling, wine, and beer industries, expired food from the confectionery and bakery industries, pomace, marc, residues from the processing of fruits and vegetables, and legume husks.

In appropriate forms, some of these can be included in a new production process as **food ingredients**. They are generally used as substances that provide a **nutritional benefit** to the consumer (rich in fiber, protein, sugars, bioactive compounds, vitamins, minerals). Their use also **reduces the environmental impact** of production processes.

Products made using ingredients that would otherwise not have been intended for human consumption are called **upcycled food**. They are produced using verifiable supply chains and have a positive impact on the environment.

Through this process, a **triple advantage** is obtained:

- At the **economic level**, giving new value to recovered raw materials at a lower cost than the original ones;
- At the **environmental level**, with less pressure on ecosystems, in the production and disposal of waste;
- At the **social level**, with a reduction in food waste.

Note: the original information was in Italian
