# Supplementary material for: Agri-Food By-Products as Ingredients: Exploring Purchase Intentions Among a Sample of Italian Consumers
Source: Foods. 2025 Jul 29;14(15):2664. doi: 10.3390/foods14152664 (PMC12345695; doi:10.3390/foods14152664)
Supplement: Supplementary file 1 [file foods-14-02664-s001.zip › File S2.pdf]

Table S1. Descriptive statistics of questionnaire items.

Item	Mean	Standard deviation	Skewness (Pearson)	Kurtosis (Pearson)
NP1 (R)	3.279	1.532	0.495	-0.625
NP2	3.242	1.543	0.425	-0.793
NP3	2.974	1.660	0.778	-0.430
NP4 (R)	2.909	1.617	0.833	-0.203
NP5	2.927	1.633	0.673	-0.580
NP6 (R)	2.626	1.426	1.084	0.448
NP7	3.170	1.778	0.514	-1.018
NP8	3.319	1.873	0.387	-1.122
NP9 (R)	2.830	1.780	0.934	-0.331
NP10	3.295	1.786	0.491	-0.870
EC1	6.038	1.215	-1.891	3.903
EC2	6.436	0.898	-2.961	11.966
EC3	5.966	1.209	-1.629	2.875
EC4	6.044	1.269	-1.991	4.053
EC5	6.069	1.077	-1.779	4.117
GP1	2.731	0.755	-0.535	0.739
GP2	3.719	0.759	-2.102	5.820
GP3	3.095	0.923	-0.810	0.726
GP4	3.830	0.562	-1.900	7.142
GP5	3.267	0.960	-1.066	1.018
FRU1	6.032	1.040	-2.063	6.258
FRU2	5.519	1.296	-1.192	1.408
FRU3	6.022	1.103	-1.910	4.849
SK1	5.014	1.758	-0.708	-0.651
SK2	4.156	1.824	-0.195	-1.167
SK3	4.436	1.827	-0.320	-1.131
SK4	3.826	1.927	-0.062	-1.324
SK5	3.527	1.909	0.248	-1.190
OK	3.622	2.189	-0.568	-1.097
ATT1	5.715	1.627	-1.224	0.750
ATT2	5.212	1.679	-0.858	0.129
ATT3	5.564	1.660	-1.091	0.494
ATT4	5.812	1.560	-1.286	0.959
PI	5.139	1.423	-1.011	0.660
WTT_P1	5.250	1.806	-0.935	0.056
WTB_P1	4.808	1.774	-0.632	-0.285
WTT_P2	5.117	2.001	-0.875	-0.369
WTB_P2	4.701	1.892	-0.574	-0.566
WTT_P3	5.238	1.887	-0.997	0.035
WTB_P3	4.857	1.856	-0.681	-0.380
WTP_P1	1.038	0.480	1.911	11.394
WTP_P2	3.341	1.220	-0.906	0.778
WTP_P3	3.658	1.200	-1.230	1.494

(R) indicated reversed items.
P1: yogurt with hazelnut skin, P2: bread with grape pomace powder; P3: biscuits with brewers' spent grains

Table S2. Results of measurement model evaluation – Reliability and validity analysis.

	First Iteration					Final Iteration				
	**Loading**	**Cronbach's α**	**CR_rho a**	**CR_rho c**	**AVE**	**Loading**	**Cronbach's α**	**CR_rho a**	**CR_rho c**	**AVE**
ATT1	0.901	0.907	0.909	0.935	0.782	0.901	0.907	0.909	0.935	0.782
ATT2	0.877					0.877				
ATT3	0.842					0.842				
ATT4	0.917					0.917				
EC1	0.787	0.799	0.802	0.862	0.556	0.787	0.799	0.802	0.862	0.556
EC2	0.819					0.819				
EC3	0.709					0.709				
EC4	0.719					0.719				
EC5	0.683					0.683				
FRU1	0.718	0.738	0.825	0.848	0.652	0.718	0.738	0.825	0.848	0.652
FRU2	0.796					0.796				
FRU3	0.899					0.899				
GP1	0.54	0.656	0.671	0.781	0.419	omitted	0.607	0.639	0.787	0.553
GP2	0.631					omitted				
GP3	0.670					0.688				
GP4	0.661					0.716				
GP5	0.72					0.82				
NP1	0.696	0.880	0.894	0.903	0.486	0.703	0.878	0.893	0.902	0.509
NP2	0.572					omitted				
NP3	0.687					0.676				
NP4	0.752					0.766				
NP5	0.7					0.696				
NP6	0.795					0.799				
NP7	0.742					0.733				
NP8	0.577					0.584				
NP9	0.575					0.590				
NP10	0.822					0.835				
OK1	0.741	0.849	0.86	0.887	0.568	0.741	0.849	0.860	0.887	0.568
OK2	0.815					0.815				
OK3	0.757					0.757				
OK4	0.737					0.737				
OK5	0.678					0.678				
OK6	0.786					0.786				
SK1	0.869	0.929	0.939	0.946	0.780	0.869	0.929	0.939	0.946	0.780
SK2	0.927					0.927				
SK3	0.922					0.922				
SK4	0.835					0.835				
SK5	0.858					0.858				

Abbreviations: AVE, average variance extracted; CR, composite reliability.
ATT: attitude, EC: environmental concern; FRU: frugality; GP: green practices; NP: food neophobia; OK: objective knowledge; SK: subjective knowledge.

Table S3. Cross loadings.

	ATT	EC	FRU	GP	NP	OK	PI	SK
ATT1	**0.901**	0.245	0.125	0.217	-0.263	0.352	0.669	0.26
ATT2	**0.877**	0.233	0.102	0.167	-0.298	0.324	0.675	0.195
ATT3	**0.842**	0.32	0.194	0.229	-0.214	0.383	0.558	0.346
ATT4	**0.917**	0.285	0.142	0.188	-0.265	0.388	0.685	0.246
EC1	0.297	**0.787**	0.331	0.335	-0.073	0.117	0.178	0.132
EC2	0.263	**0.819**	0.298	0.283	-0.143	0.11	0.186	0.088
EC3	0.195	**0.709**	0.213	0.182	-0.063	0.057	0.165	0.084
EC4	0.187	**0.719**	0.25	0.279	-0.138	0.063	0.142	0.073
EC5	0.186	**0.683**	0.209	0.218	-0.049	0.097	0.196	0.14
FRU1	0.127	0.256	**0.718**	0.212	-0.089	0.097	0.091	0.128
FRU2	0.102	0.259	**0.796**	0.362	0.027	0.04	0.088	0.201
FRU3	0.149	0.323	**0.899**	0.347	-0.047	0.092	0.15	0.146
GP3	0.113	0.249	0.359	**0.631**	-0.046	0.145	0.14	0.206
GP4	0.133	0.243	0.314	**0.661**	-0.059	0.13	0.124	0.164
GP5	0.146	0.144	0.271	**0.72**	-0.049	0.137	0.202	0.151
NP1	-0.188	-0.123	-0.04	-0.087	**0.696**	-0.137	-0.222	-0.188
NP3	-0.208	-0.035	0.041	-0.001	**0.687**	-0.109	-0.201	-0.046
NP4	-0.237	-0.118	-0.089	-0.139	**0.752**	-0.102	-0.263	-0.088
NP5	-0.215	-0.059	-0.054	-0.103	**0.7**	-0.128	-0.198	-0.048
NP6	-0.237	-0.152	-0.06	-0.155	**0.795**	-0.102	-0.292	-0.09
NP7	-0.184	-0.046	0.021	-0.068	**0.742**	-0.123	-0.205	-0.017
NP8	-0.139	0.017	-0.046	-0.01	**0.577**	-0.068	-0.149	-0.092
NP9	-0.159	-0.028	-0.063	-0.044	**0.575**	-0.133	-0.186	-0.206
NP10	-0.254	-0.144	-0.059	-0.128	**0.822**	-0.102	-0.301	-0.119
OK1	0.332	0.1	0.068	0.178	-0.113	**0.741**	0.269	0.423
OK2	0.353	0.112	0.071	0.173	-0.148	**0.815**	0.311	0.462
OK3	0.258	0.147	0.145	0.157	-0.12	**0.757**	0.201	0.45
OK4	0.368	0.075	0.064	0.172	-0.15	**0.737**	0.311	0.383
OK5	0.205	0.036	0.029	0.096	-0.046	**0.678**	0.213	0.393
OK6	0.269	0.079	0.069	0.143	-0.13	**0.786**	0.227	0.479
PI	0.733	0.236	0.142	0.235	-0.324	0.349	1	0.276
SK1	0.284	0.126	0.201	0.235	-0.125	0.576	0.259	**0.869**
SK2	0.258	0.138	0.187	0.24	-0.078	0.515	0.231	**0.927**
SK3	0.302	0.144	0.208	0.238	-0.147	0.508	0.281	**0.922**
SK4	0.219	0.125	0.13	0.205	-0.115	0.466	0.222	**0.835**
SK5	0.22	0.087	0.091	0.208	-0.131	0.438	0.213	**0.858**

ATT: attitude, EC: environmental concern; FRU: frugality; GP: green practices; NP: food neophobia; OK: objective knowledge; SK: subjective knowledge.
